# A Comparison of Third-Generation Semi-Invasive Arterial Waveform Analysis with Thermodilution in Patients Undergoing Coronary Surgery

**DOI:** 10.1100/2012/451081

**Published:** 2012-07-31

**Authors:** Ole Broch, Jochen Renner, Matthias Gruenewald, Patrick Meybohm, Jan Schöttler, Markus Steinfath, Manu Malbrain, Berthold Bein

**Affiliations:** ^1^Department of Anaesthesiology and Intensive Care Medicine, University Hospital Schleswig-Holstein, 24105 Kiel, Germany; ^2^Department of Anaesthesiology, Intensive Care Medicine and Pain Therapy, University Hospital Frankfurt, 60590 Frankfurt am Main, Germany; ^3^Department of Cardiothoracic and Vascular Surgery, University Hospital Schleswig-Holstein, 24105 Kiel, Germany; ^4^ICU and High Care Burn Unit, ZNA, Campus Stuivenberg, 2060 Antwerpen, Belgium

## Abstract

Uncalibrated semi-invasive continous monitoring of cardiac index (CI) has recently gained increasing interest. The aim of the present study was to compare the accuracy of CI determination based on arterial waveform analysis with transpulmonary thermodilution. Fifty patients scheduled for elective coronary surgery were studied after induction of anaesthesia and before and after cardiopulmonary bypass (CPB), respectively. Each patient was monitored with a central venous line, the PiCCO system, and the FloTrac/Vigileo-system. Measurements included CI derived by transpulmonary thermodilution and uncalibrated semi-invasive pulse contour analysis. Percentage changes of CI were calculated. There was a moderate, but significant correlation between pulse contour CI and thermodilution CI both before (*r* ^2^ = 0.72, *P* < 0.0001) and after (*r* ^2^ = 0.62, *P* < 0.0001) CPB, with a percentage error of 31% and 25%, respectively. Changes in pulse contour CI showed a significant correlation with changes in thermodilution CI both before (*r* ^2^ = 0.52, *P* < 0.0001) and after (*r* ^2^ = 0.67, *P* < 0.0001) CPB. Our findings demonstrated that uncalibrated semi-invasive monitoring system was able to reliably measure CI compared with transpulmonary thermodilution in patients undergoing elective coronary surgery. Furthermore, the semi-invasive monitoring device was able to track haemodynamic changes and trends.

## 1. Introduction

Estimation of haemodynamic variables such as left ventricular stroke volume and cardiac index in high-risk patients is a prerequisite for performing individual goal-directed therapy. Perioperative haemodynamic optimization and individual tailored therapy have been shown to improve patients' outcome by reducing morbidity and the length of stay on the intensive care unit [1–3]. In clinical practice, determination of cardiac index in the past was mostly related to invasive procedures such as right heart catheterization or femoral access, baring method-related complications and limitations [4–6]. In this context, less-invasive techniques based on arterial waveform analysis have gained increasing interest [7–9]. An example for a semi-invasive device for estimation of CI by pulse contour analysis is the FloTrac/Vigileo-system (Edwards Lifesciences LLC, Irvine, CA, USA), which was introduced in 2005 and since then underwent several software upgrades. The underlying method is described in detail elsewhere [10, 11]. This system requires only an arterial line connected to a special transducer (FloTrac) and has been investigated in several studies under various clinical conditions, but its precision to reflect CI is still under debate [12–14].

The aim of the present study was to investigate the precision of CI determination based on arterial waveform analysis by a third-generation device (CI_Wave_) with transpulmonary thermodilution (CI_TPTD_) before and after cardiopulmonary bypass (CPB). Furthermore, we studied the ability of the semi-invasive technique for tracking haemodynamic changes and trends.

## 2. Materials and Methods

After approval from our institutional ethics committee (Christian Albrecht University Kiel, Schwanenweg 20, D-24105 Kiel), written informed consent for participation in the study was obtained from all patients.

Fifty patients undergoing elective coronary artery bypass grafting (CABG) were studied after induction of general anaesthesia until discharge to the intensive care unit. Exclusion criteria were patients <18 years of age, a left ventricular ejection fraction ≤0.5, emergency procedures, and patients with haemodynamic instability requiring continuous pharmacologic support. Patients with intracardiac shunts, severe aortic, tricuspid or mitral stenosis or insufficiency and mechanical circulatory support were also excluded.

### 2.1. Instrumentation and Protocol

All patients were premedicated with midazolam 0.1 mg/kg orally 30 min before induction of anaesthesia. Routine monitoring was established including peripheral oxygen saturation (SpO_2_) and heart rate (HR) (S/5 monitor, GE Healthcare, Helsinki, Finland). Subsequently patients received a peripheral venous access and a radial arterial line. A FloTrac/Vigileo-system was connected to the arterial line, followed by adjustment of the transducer and input of required individual demographic data according to the manufacturer's instructions (Software version 1.07, “third generation”). Variables were automatically indexed to body surface area. After induction of anaesthesia with sufentanil (0.5 *μ*g/kg) and propofol (1.5 mg/kg), orotracheal intubation was facilitated with rocuronium (0.6 mg/kg). Anaesthesia was maintained with sufentanil (1 *μ*g/kg/h), and propofol (3 mg/kg/h) and patients were ventilated with an oxygen/air mixture using a tidal volume of 8 mL/kg ideal body weight and positive end-expiratory pressure was set at 5 cm H_2_O. After placement of a central venous catheter in the right internal jugular vein, a transpulmonary thermodilution catheter (Pulsion Medical Systems; Munich, Germany) was introduced in the femoral artery. The thermodilution catheter was connected to the PiCCO_2_ monitor (Software version 1.3.0.8).

### 2.2. Data Collection

**Figure 1:**
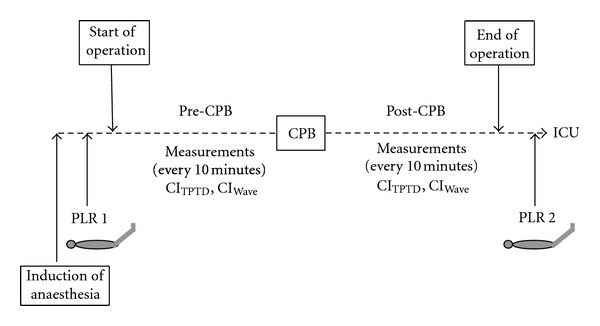
Study design. T1: data collection (CI_Wave_, CI_TPTD_) after induction of anaesthesia and passive leg raising (PLR 1) until cardiopulmonary bypass; T2: data collection (CI_Wave_, CI_TPTD_) after cardiopulmonary bypass until the end of surgery and passive leg raising (PLR 2).

After induction of anaesthesia, CI_TPTD_ and CI_Wave_ were recorded every 10 minutes both before and after CPB. Stable haemodynamic conditions and an undamped arterial signal were prerequisites for the measurements. CI_TPTD_ was determined by injecting 15 mL ice cold saline (≤8°C) through the central venous line at least three times randomly assigned to the respiratory cycle. In case of a difference of ≥15%, the value obtained was discarded and the measurement repeated. Simultaneously, measurement of CI_Wave_ was performed by plotting 5 numerical values over a period of three minutes and determining the mean value. Systolic, diastolic, and mean arterial pressures as well as CVP were also recorded every 10 minutes. After induction of anaesthesia, a passive leg raising manoeuvre (PLR 1) was performed, and haemodynamic variables including CI (CI_TPTD_, CI_Wave_) and stroke volume index (SVI) were recorded before, during and after PLR. Subsequently, measurements of CI_TPTD_ and CI_Wave_ were carried out every 10 minutes until the beginning of CPB (T1), which differed from patient to patient yielding different numbers of measurements in this time period. Measurements of CI_TPTD_ and CI_Wave_ were restarted 15 minutes after weaning from CPB and were obtained up to the end of the surgical intervention (T2), again yielding a different number of measurement pairs in individual patients. Immediately before discharge to the intensive care unit, a second PLR manoeuvre (PLR 2) was performed. Study design is displayed in Figure 1.

### 2.3. Statistical Analysis

All data are given as mean ± SD. Statistical comparisons were performed using commercially available statistics software (GraphPad Prism 5, GraphPad Software Inc., San Diego, CA, USA, and MedCalc for Windows, version 11.6.1.0, MedCalc Software, Mariakerke, Belgium). To demonstrate the relationship between sample size and the width of the confidence interval of the estimated variable, we calculated the width of the 95% confidence interval of the limits of agreement as recommended by Bland and Altman (as ±1.96$\sqrt{\left(3/n\right)}\cdot s$, where *s* is the standard deviation of the bias). For our sample size of 50 patients, we calculate a width of the 95% confidence interval of the limits of agreement of 0.51 times the SD (of bias), which is generally thought to be an acceptable narrow 95% confidence interval.

To describe the agreement between CI_TPTD_ and CI_Wave_, Bland-Altman plots for repeated measures were calculated for each time period (T1-T2) before and after CPB. Percentage error was calculated as described by Critchley and colleagues, using the limits of agreement (2SD) of the bias divided by the mean CI values from CI_TPTD_ and CI_Wave_. Bland-Altman plots were also performed for haemodynamic trends (ΔCI_TPTD_, ΔCI_Wave_) before and after CPB. Changes of CI_TPTD_ < 15% were excluded from analysis as recommended by Critchley and colleagues [15]. Unpaired sample *t*-test was used to analyse significant differences of arterial pressure and systemic vascular resistance index (SVRI) related to the periods of measurement.

## 3. Results

Data of all 50 patients, 37 males and 13 females, were included into final analysis. Age ranged between 40 and 85 years, with a mean age of  63 ± 5  and a mean body mass index of 26.8 ± 3.5 kg·m^−2^. Mean left ventricular ejection fraction was 0.61 ± 0.07%. A total of 468 data pairs (T1: 245, T2: 223) were obtained during the study period. Haemodynamic variables are shown in Table 1.

**Figure 2:**
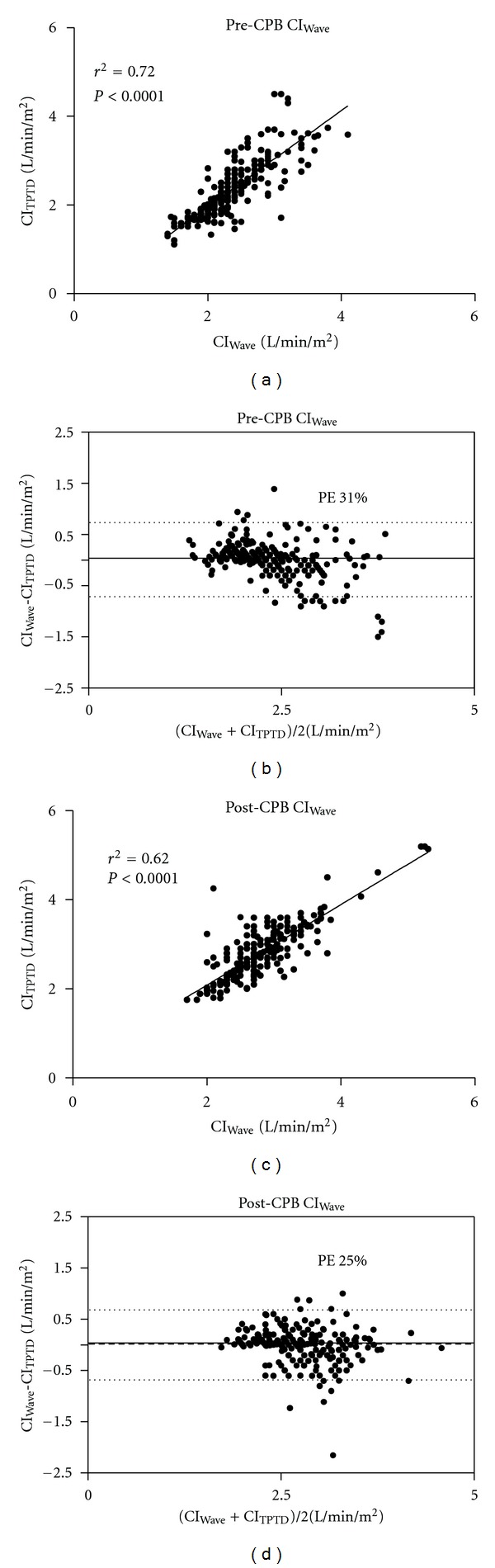
Correlation and Bland-Altman analysis of cardiac index measured by transpulmonary thermodilution (CI_TPTD_) and cardiac index measured by uncalibrated semi-invasive pulse contour analysis (CI_Wave_) before (T1) and after (T2) cardiopulmonary bypass.

There was a significant correlation between CI_Wave_ and CI_TPTD_ before (T1) and after (T2) cardiopulmonary bypass (Figure 2). Correlations, bias, LOAs, and percentage errors for each time period (T1-T2) are summarized in Table 2. PLR manoeuvre before CPB (PLR 1) was performed in 47/50 patients and in 42/50 patients after CPB (PLR 2), respectively. Patients who increased their SVI_TPTD_ > 15% during PLR were defined as responders. We observed 24 responders during PLR 1 (51%) and 23 responders during PLR 2 (55%), respectively. There was a significant correlation between CI_Wave_ and CI_TPTD_  (*r* ^2^ = 0.76, *P* < 0.0001) at PLR 1. Correlations and Bland Altman analysis with bias, LOAs, and percentage errors for PLR before (PLR 1) and after (PLR 2) CPB are presented in Table 2.

**Figure 3:**
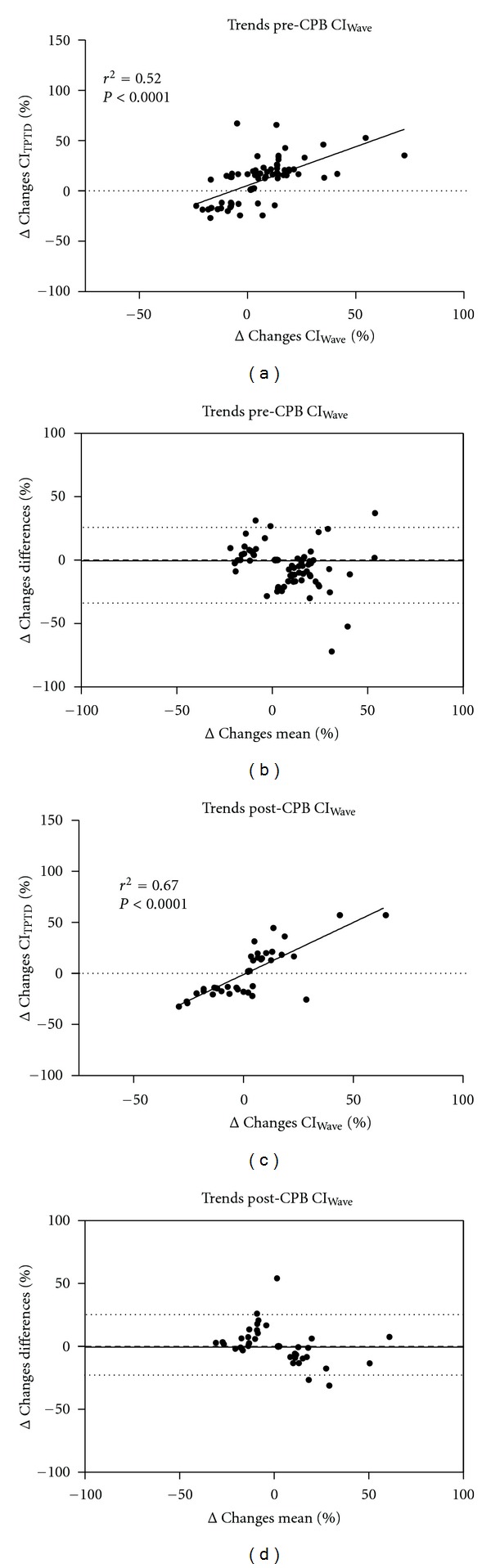
Correlation and Bland-Altman analysis of percentage changes in cardiac index measured by transpulmonary thermodilution (ΔCI_TPTD_) and cardiac index measured by uncalibrated semi-invasive pulse contour analysis (ΔCI_Wave_) before and after cardiopulmonary bypass (CPB).

The percentage changes for ΔCI_Wave_ versus ΔCI_TPTD_ are presented in Figure 3. Bland-Altman analysis showed a significant correlation for ΔCI_Wave_ versus ΔCI_TPTD_ in T1, with LOAs from −34% to +26%. After CPB (T2), correlation coefficients of changes in ΔCI_Wave_ versus ΔCI_TPTD_ again were statistically significant, with LOAs ranging from −23 to +25% (Figure 3). There was a significant relationship between CI_Wave_ and SVRI before (CI_Wave_: *r* ^2^ = 0.27, *P* < 0.0001) and after (CI_Wave_: *r* ^2^ = 0.12, *P* < 0.0001) CPB. No significant correlation between MAP and CI_Wave_ was observed before (CI_Wave_: *r* ^2^ = 0.003, *P* = 0.42) and after (CI_Wave_: *r* ^2^ < 0.006, *P* = 0.25) CPB. Unpaired *t*-test showed a significant difference between SVRI before and after CPB (*P* < 0.05).

## 4. Discussion

Main findings of the present study are that the semi-invasive monitoring device was able to reliably measure CI compared with transpulmonary thermodilution before and after CPB. There was a weak but significant correlation between semi-invasive CI by arterial waveform analysis and systemic vascular resistance index. The semi-invasive monitoring system was able to track haemodynamic changes and trends before and after CPB.

The semi-invasive FloTrac/Vigileo-system was developed to continuously determine CI and stroke volume by arterial waveform analysis without the need for calibration. As described elsewhere [11], this system is based on a special software algorithm which calculates CI continuously by analysis of the arterial blood pressure tracing. Before starting the system, patient specific data like age, gender, and body surface area are required. With respect to the three major vascular properties: impedance, compliance, and resistance, determination of cardiac output from arterial waveform analysis is also influenced by the inverse relationship between aortic diameter and pressure [16, 17]. This relationship is reflected in the FloTrac/Vigileo-algorithm by using several variables like demographic data, mean arterial pressure, and shape of the arterial waveform to calculate an essential part of the algorithm: a special equitation named chi (*χ*). With respect to rapid changes in vascular tone, the software version investigated in the present study is able to recalculate *χ* every 60 seconds.

Monitoring of haemodynamic variables is mostly related to invasive procedures, sometimes time-consuming, difficult to establish and associated with method-related complications and limitations, respectively [4]. Therefore, a semi-invasive, quick available, and easy to install method for continuous estimation of haemodynamic variables may be advantageous for the clinician in the decision making process of patients' therapy. Furthermore, besides determination of absolute values of CI, accurate tracking of haemodynamic trends could also be extremely valuable while performing goal-directed therapy. However, sufficient accuracy of a monitoring system is a prerequisite before use in daily clinical routine. To date, several studies investigated the semi-invasive monitoring device in varying clinical conditions exhibiting equivocal results [12, 14]. We studied the “third-generation” software in patients undergoing elective coronary artery surgery and during a passive leg raising manoeuvre both before and after cardiopulmonary bypass.

Applying the criteria as recommended by Critchley and colleagues [18], we regarded the semi-invasive device as interchangeable with the reference technique if the percentage error did not exceed 30%. Before bypass, the semi-invasive device narrowly missed the strict requirements as recommended by these authors. These limits, however, have been criticized extensively by recent literature due to lack of determination of precision and nonconsideration of haemodynamic trends [19]. In this context, recent literature demonstrated less accuracy of CI by arterial waveform analysis in presence of low vascular resistance [12, 13, 20]. Accordingly, we observed a weak correlation between CI measured by semi-invasive arterial waveform analysis and systemic vascular resistance index both before and after cardiopulmonary bypass. It must be noted, however, that this observation is based on only few data points from a small number of patients. Interestingly, we observed interchangeable results for CI generated by semi-invasive monitoring device and transpulmonary thermodilution especially after cardiopulmonary bypass; a study period typically exhibiting lower values of systemic vascular resistance index. An explanation of these conflicting results may be that in one of the studies cited above patients with septic shock were enrolled, certainly presenting different systemic vascular resistance indices compared to our patients after cardiopulmonary bypass [12]. Furthermore, from a clinical point of view, CI will be higher in presence of lower systemic vascular resistance. These findings will explain the relationship found in our study, especially since we obtained interchangeability between arterial waveform analysis and transpulmonary thermodilution. With respect to other variables influencing vascular tone, several studies demonstrated failure of arterial waveform analysis in presence of vasoactive agents or high mean arterial pressure [21–23]. Interestingly, we did not observe a relationship between CI by semi-invasive arterial waveform analysis and mean arterial pressure. To investigate the ability of the semi-invasive device for tracking rapid changes in CI even in presence of lower systemic vascular resistance, a passive leg raising maneouvre was performed both before and after cardiopulmonary bypass. We obtained a significant correlation between CI by arterial waveform analysis and CI by transpulmonary thermodilution during the passive leg raising maneouvres. During the first manoeuvre, the semi-invasive monitoring system failed by a narrow margin to achieve interchangeability with the reference technique, but after cardiopulmonary bypass accuracy was sufficient. However, beside determination of absolute values of CI, a monitoring system should be able to track haemodynamic trends to reflect therapeutic interventions. Therefore, we calculated trends in CI for both monitoring systems and excluded changes of CI obtained by transpulmonary thermodilution <15% from further analysis as recommended by recent literature [15]. The semi-invasive device showed a good ability for following trends before and after cardiopulmonary bypass.

Some limitations of our study should be noted. We investigated patients with preserved left ventricular function undergoing elective coronary artery surgery without ongoing pharmacologic support, and we excluded patients with haemodynamic instability or in shock. Therefore, our results cannot be directly transferred to patients with impaired left ventricular function or inotropic and vasoactive support.

With respect to clinical relevance, several studies could demonstrate that an individual tailored therapy was associated with reduced perioperative morbidity. Determination of cardiac index continuously is an important target while performing goal-directed therapy.

In conclusion, the present study could demonstrate reliable measurement of cardiac index and haemodynamic trends by semi-invasive arterial waveform analysis in patients undergoing elective coronary surgery and with preserved left ventricular function. Therefore, our results support the use of less invasive haemodynamic monitoring systems.

## Figures and Tables

**Table 1:** Haemodynamic variables before (T1) and after (T2) cardiopulmonary bypass.

Variables	Pre-CPB	Post-CPB	
	T1 (*n* = 245)	T2 (*n* = 223)	*P*
HR (min^−1^)	57 ± 3	80 ± 4	*P* < 0.05^#^
MAP (mmHg)	76 ± 5	73 ± 10	*P* < 0.05^#^
CVP (mmHg)	10 ± 2	9 ± 4	*P* = 0.11
SVRI (dynes·s/cm^5^/m^2^)	2370 ± 62	1966 ± 121	*P* < 0.05^#^
CI_TPTD_ (L/min/m²)	2.4 ± 0.6	2.8 ± 0.6	*P* < 0.05^#^
CI_Wave_ (L/min/m²)	2.4 ± 0.5	2.8 ± 0.6	*P* < 0.05^#^

CPB: cardiopulmonary bypass; HR: heart rate; MAP: mean arterial pressure; CVP: central venous pressure; SVRI: systemic vascular resistance index; CI_TPTD_: cardiac index by transpulmonary thermodilution; CI_Wave_: cardiac index by semi-invasive pulse contour analysis; values are given as mean ± SD. ^#^ *P* < 0.05 (versus T1).

**Table 2:** Bland-Altman analysis showing 95% limits of agreement, confidence interval, and percentage error before (T1) and after (T2) cardiopulmonary bypass and during passive leg raising before (PLR 1) and after (PLR 2) bypass.

	T1	T2	PLR 1	PLR 2
*n*_data_/*n*_patient_	*n* = 245/*n* = 50	*n* = 223/*n* = 50	*n* = 132/*n* = 47	*n* = 123/*n* = 42
	CI_Wave_	CI_Wave_	CI_Wave_	CI_Wave_
Mean (L/min/m^2^)	2.38	2.78	2.26	2.76
Bias (L/min/m^2^)	0.01	0.007	0.05	0.03
SD of bias (L/min/m^2^)	0.37	0.35	0.34	0.34
CI of LOA (L/min/m^2^)	0.17	0.16	0.10	0.11
95% limits of agreement (L/min/m^2^)	−0.71 to +0.73	−0.69 to +0.68	−0.63 to +0.72	−0.69 to +0.63
Percentage error (%)	31	25	30	25

CI_Wave_: cardiac index by semi-invasive pulse contour analysis; CI_TPTD_: cardiac index by transpulmonary thermodilution; CI of LOA: confidence interval of the limits of agreement; PLR: passive leg raising.
